# Adhesion Properties of Recycled High-Viscosity Asphalt–Aggregate Interface under Dynamic Water Erosion

**DOI:** 10.3390/ma16186203

**Published:** 2023-09-14

**Authors:** Kang Zhao, Shijie Song, Yang Wei, Guofen Li, Feng Guo

**Affiliations:** College of Civil Engineering, Nanjing Forestry University, Nanjing 210037, China; zkas@njfu.edu.cn (K.Z.); shijiesong@njfu.edu.cn (S.S.); 18816210278@163.com (F.G.)

**Keywords:** drainage asphalt pavement, recycled high-viscosity asphalt, dynamic water erosion, ultrasonic numerical simulations, AFM, adhesion performance

## Abstract

The drainage of asphalt pavement requires the use of a large amount of high-viscosity-modified asphalt, which faces the service environment under dynamic water erosion. The feasibility of recycling high-viscosity-modified asphalt should be investigated to facilitate sustainable infrastructure construction. This study used ultrasonic equipment to simulate dynamic water erosion test conditions and tested the adhesion performance of different types of recycled high-viscosity asphalt at various environmental temperatures. The adhesion energy index and microstructure of recycled high-viscosity asphalt were analyzed using the contact angle test and AFM test. The results demonstrate that the higher the environmental temperature, the worse the anti-stripping performance of recycled high-viscosity asphalt. From the perspective of adhesion performance indicators, a 6% recycling agent dosage is more conducive to restoring the performance of aged high-viscosity -modified asphalt. The AFM test showed that the microstructure of high-viscosity -modified asphalt represented significant changes with an increase in the recycling agent content, and the change in the adhesion force of recycled high-viscosity -modified asphalt was consistent with the results of macroscopic adhesion performance tests. This study illustrates the applicability of implementing regeneration technology for the recycling of aged drainage asphalt pavement.

## 1. Introduction

In order to ensure the application effect of drainage asphalt pavement, researchers have developed different types of phase change materials to improve the high-temperature stability of the mixture on the one hand and used a high-viscosity asphalt binder to improve the overall road performance of the mixture on the other hand [1,2,3,4,5,6]. Generally speaking, improving asphalt viscosity can be realized by adding modifiers, among which Tafpack Super(TPS) high-viscosity-modified asphalt has been widely used because of its excellent high-temperature stability, low-temperature crack resistance, strong cohesion, and durability [7]. However, although higher viscous asphalt with better performance is used to drain asphalt pavements, due to the design concept of an open-graded and large void ratio (18–25%), pavements still have the disadvantage of rapid decay in surface function, low strength, and poor durability, and are prone to diseases such as void blockage, water damage, and fatigue cracking during service, and therefore, must be maintained periodically [8].

In China, up to 70 million tons of waste asphalt pavement materials are produced annually, and while the basalt aggregate used for the drainage of asphalt pavement has low reserves and the high-viscosity-modified asphalt cost is high, the use of recycled technology for pavement rehabilitation is particularly necessary [9,10,11,12]. On the one hand, researchers used the recycling agent to regenerate aged high-viscosity asphalt and evaluate the conventional performance of recycled asphalt. On the other hand, the mechanical properties and durability of recycled drainage asphalt mixtures were investigated at different old material mixing levels (15–93%) [13]. Xu et al. [14] developed a naphthenic asphalt rejuvenator for drainage asphalt pavements, which could improve the permeability and ductility of aged high-viscosity asphalt; however, the adhesion between asphalt and aggregate is insufficient, and asphalt can easily peel off from the surface of the aggregate in a humid environment. Frigio et al. [13] found that the use of an asphalt-based recycling agent could improve the strength and flexibility of recycled drainage asphalt mixtures, but the enhancement of its resistance to water damage was very limited. The road performance of reclaimed drainage asphalt mixtures with 30% old material basically meets the specification requirements, but its water stability is slightly poor, and its long-term performance needs to be further observed [15]. At present, recycled high viscous asphalt–aggregate interface adhesion performance research is limited, and the high viscous asphalt regeneration effect and adhesion performance recovery law have to be studied.

For vehicles traveling on the road surface, road surface water is under the action of the vehicle load constantly in the drainage of an asphalt mixture between the gap in the back and forth scouring, constantly abrading the asphalt in the connecting part between the aggregate and the aggregate, and ultimately resulting in the loss of asphalt’s adhesion ability. It has been shown that the dynamic water action caused by vehicle loading is the main cause of water damage in asphalt mixtures, which is particularly significant in drained asphalt pavements [16]. Due to the huge difference between the hydrostatic test in the laboratory and the actual road used, many reclaimed roads experience different degrees of water damage within a short period of time after being put into operation. Therefore, in order to better investigate the scouring effect of vehicle loads on the asphalt–aggregate interface in the presence of water, simulation tests need to be designed in the laboratory [17,18]. A dynamic water pressure experimental simulator was designed for the test using the idea of the pneumatic pressure approach, and the final results obtained show that the main reason for the attenuation of the mechanical properties of asphalt mixtures is a decrease in asphalt viscosity under the condition that the dynamic water scour is directional and quantitative [19]. An ultrasonic dynamic water scour simulation device was used to simulate the scouring effect of water on the asphalt–aggregate interface in the open road and was combined with the image processing method to realize the quantitative description of the adhesion properties of the asphalt–aggregate interface [20]. The ultrasonic dynamic water scour simulation device is simple, and the ultrasonic cavitation phenomenon can simulate the extrusion and dynamic water scouring effect of the vehicle load on the road surface well on rainy days, which is especially suitable for the drainage of asphalt pavement.

In this paper, an ultrasonic dynamic water scour simulation device was used to study the effects of the high-viscosity agent dosage, recycling agent dosage, and temperature on the asphalt spalling rate on the aggregate surface and to analyze the anti-stripping performance of recycled high-viscosity asphalt under the dynamic water scour condition. The surface energies of asphalt and aggregate were measured and calculated using the lay-drop method, and the adhesion properties of a recycled high viscous asphalt–aggregate interface were evaluated using the parameters of the spreading coefficient, work of adhesion, and the work of spalling and energy ratio. An Atomic Force Microscope (AFM) (IBM, Armonk, NY, USA) was used to obtain the surface topography, force curve, and other microscopic information from the recycled high viscous asphalt to study the effects of the high viscous agent dosage and recycling agent dosage on the honeycomb structure, roughness, and adhesion force of asphalt, and to reveal the mechanism of changes to the adhesion properties of the recycled high viscous asphalt.

## 2. Materials Preparation and Test Methods

### 2.1. Materials

The technical indicators described in this paper for the study of matrix asphalt and which Alpha (Jiangyin) Asphalt Co., Ltd. (Nanjing, China) used to provide the ordinary 70# matrix asphalt are shown in Table 1. A high-viscosity modifier was selected for commonly used TPS materials the shape of the particle size was about 5 mm in the form of dark brown spherical particles and could be melted at 130 °C or more in the asphalt. The regeneration agent was the Anshan Shuangcheng brand asphalt regeneration agent, which is a yellow-brown liquid at room temperature. In this paper, a basalt aggregate above a 13.2 mm screen and below a 16 mm screen was obtained through the sieving test as the coarse aggregate used in the adhesion performance test.

### 2.2. Preparation of Recycled High-Viscosity Modified Asphalt

Since the TPS dosage varies in different projects: the dosage of a high-viscosity modifier was formulated as 10%, 12%, and 14% in this paper. In this paper, high-viscosity-modified asphalt was prepared using the shear method; first, the 70# base asphalt was heated to 140 °C and kept at a constant temperature; then, a specified dosage of the high-viscosity modifier was added and stirred evenly with a glass rod; finally, a high-speed shear was used to continuously shear for 30 min at a rate of 4000 r/min until the modifier was uniformly fused in the asphalt, and high-viscosity modified asphalt was obtained.

According to the “Standard test methods of bitumen and bituminous mixtures for highway engineering” (JTG E20-2011) in the T 0609-2011 and T 0630-2011 preparation of aging high-viscosity asphalt [21], at each time, 50 g of prepared high-viscosity asphalt was poured into the metal specimen dish in the film oven (TFOT) at 163 °C under the condition of 5 h of simulated asphalt with short-term aging. Short-term aging occurred after the end of the further in the pressure aging vessel (PAV) in a 100 °C and 2.1 MPa environment to maintain 20 h of simulated asphalt long-term aging.

In order to study the recovery effect of different rejuvenating agent dosages on the adhesive properties of aged high viscous asphalt, the aged high viscous asphalt was heated to a constant temperature of 170 °C, and then different ratios of the rejuvenating agent (4%, 6% and 8%) were added, and the rejuvenated high viscous asphalt was obtained using high-speed shear to mix at a rate of 2000 r/min for 30 min. The performance parameters for different types of recycled high-viscosity asphalt were measured, the results of which are shown in Table 2, and the recycled high-viscosity asphalt type named rules for R-high-viscosity modifier dosing-recycling agent dosing.

The preliminary analysis of the needle penetration index can be seen in the same regeneration agent dosage with an increase in the high-viscosity agent dosage; the needle penetration roughly showed a gradual decline in the trend, indicating that the amount of high-viscosity agent is directly proportional to the consistency of recycled high-viscosity asphalt. This is due to the TPS high-viscosity modifier as a thermoplastic elastomer where high temperatures and asphalt with full shear mixing occur in the fusion and dissolution, resulting in recycled high-viscosity asphalt in the components of the change, and the force between molecules increases, exhibiting high consistency and reduced fluidity [22]. Under the condition of the same high-viscosity agent dosage with an increase in the dosage of the reclaiming agent, the needle penetration roughly showed a gradual increase in the trend, indicating that the dosage of the reclaiming agent is inversely proportional to the consistency of recycled high-viscosity asphalt. This is due to the fact that the main component of the rejuvenating agent is light oils, which can supplement the aging of asphalt in missing saturated and aromatic components, thus reducing the consistency of recycled high-viscosity asphalt, increasing rheology as well as needle penetration.

Taking the softening point as the analysis index, it can be found that under the condition of the same regenerant doping, the softening point increased with the increase in high-viscosity agent doping. Taking 4% regenerant doping as an example, the softening point increased from 63.3 °C to 78.8 °C with the increase in high-viscosity agent doping from 10% to 14%, and this increase was obvious. Taking the high-viscosity agent dosage of 10% as an example, with the increase in recycling agent dosage, the softening point of recycled high-viscosity asphalt decreases. Ductility is the main index to evaluate the plasticity of asphalt. The inclusion of a high-viscosity agent on asphalt ductility has a certain weakening effect, and the smaller the dosage of its ductility, the worse its plasticity is. On the contrary, the dosage of the regeneration agent has a certain enhancement effect on asphalt ductility.

### 2.3. Experimental Methods

#### 2.3.1. Adhesion Test

In this paper, the YM-040PLUS (Fangao Microelectronics Co., Ltd., Shenzhen, China) ultrasonic generator was used for the test; the maximum capacity of the machine was 10 L, there were 6 ultrasonic vibrators, the heating power was 300 W, the ultrasonic power was 360 W, and the ultrasonic frequency was 40 KHz. Combined with the experimental experience of the predecessors, this paper set the dynamic water scouring time as 90 min in order to simulate the drained asphalt pavement in the harsh water damage environment in service for 3 years [20]. This study used this instrument to conduct ultrasonic asphalt aggregate adhesion tests on different types of recycled high-viscosity asphalt at temperatures of 20 °C, 50 °C, and 80 °C with reference to the test steps provided in reference [23]. Three parallel trials were conducted for each test condition. The spalling rate was used as the evaluation index to analyze the effects of three factors, namely, temperature, high-viscosity agent dosage, and recycling agent dosage, on the adhesion performance of a recycled high-viscosity asphalt–aggregate interface.

#### 2.3.2. Contact Angle Test

(1) Test methods

This test was carried out using a KRUSSS-type contact angle tester (KRÜSS Scientific Instruments Co., Ltd., Hamburg, Germany), the main parts of which include an optical system, a sample conditioner, a video acquisition system, and an image analysis system, with a measurement range of 5° to 180° and an accuracy of ±0.1° [24]. Calculating the surface energy of asphalt and aggregate requires three liquids with known surface-free energy and its components. In this paper, distilled water, formamide, and glycerol were selected as the probing liquids, and the contact angles of the liquids on different recycled high-viscosity asphalt and aggregates were measured using the lay-drop method. Three parallel tests under equal conditions were performed for each measurement.

(2) Computational modeling

Surface energy is the work required to produce a new interface per unit area of a given material in a vacuum. It is known from the analysis in the literature that the surface energy of a substance (γ) is mainly composed of a polar component ($\gamma^{AB}$) and the dispersion component ($\gamma^{LW}$), while the polar component includes Lewis acid ($\gamma^{+}$) and Lewis base ($\gamma^{-}$), whose relationship is shown in Equation (1) [25].

(1) $$\gamma =\gamma^{LW}+\gamma^{AB}=\gamma^{LW}+2\sqrt{\gamma^{+}\gamma^{-}}$$

According to the surface physicochemical theory, the liquid wraps and adheres to the solid surface, and the surface energy change in the solid–liquid system is expressed by Equation (2):

(2) $$W_{ls}=\gamma_{l}+\gamma_{s}-\gamma_{ls}$$

In this Equation, $W_{ls}$ is the liquid-solid adhesion work, $\gamma_{l}$ is the liquid surface energy, $\gamma_{s}$ is the solid surface energy, and $\gamma_{ls}$ is the interfacial energy on the contact surface of the solid–liquid system.

The free energy of the interface between a liquid and a solid is expressed by Equation (3):

(3) $$\gamma_{ls}=\gamma_{l}+\gamma_{s}-2\sqrt{\gamma_{l}^{AB}\gamma_{s}^{AB}}-2\sqrt{\gamma_{l}^{LW}\gamma_{s}^{LW}}$$

In this Equation, $\gamma_{l}^{LW}$ and $\gamma_{s}^{LW}$ are the dispersion components for liquids and solids, respectively, while $\gamma_{l}^{AB}$ and $\gamma_{s}^{AB}$ are the polar components of the liquid and solid, respectively.

According to Young’s equation, the relationship shown in Equation (4) is satisfied between the contact angle of a liquid with a solid surface and its surface-free energy and interfacial energy:

(4) $$\gamma_{l}cos\theta =\gamma_{s}-\gamma_{ls}$$

In this Equation, θ is the contact angle between the liquid and the solid.

Combining Equations (1)–(4) yields the Young–Dupre formula (Equation (5)):

(5) $$\;\;\;\;\;\;\;\;\;\;\;\;\;\;\;\;\;\;W_{ls}=\gamma_{l}\left(1+cos\theta \right)=2\sqrt{\gamma_{l}^{LW}\gamma_{s}^{LW}}+2\sqrt{\gamma_{l}^{+}\gamma_{s}^{-}}+2\sqrt{\gamma_{l}^{-}\gamma_{s}^{+}}$$

At room temperature, the asphalt is a solid. By measuring the contact angles between three types of liquids with known surface energy parameters and solid asphalt surfaces, a system of equations can be established according to Equation (5) to obtain the surface-free energy component value of asphalt before substituting it into Formula (1), which can be calculated from the surface free energy of asphalt where the solid surface energy calculation is the same [26].

(3) Evaluation indicators
  a. Spreading factor

Wetting is the process by which a liquid droplet on a solid surface achieves contact with the solid and continues to spread. It can be seen that wettability can be used to evaluate the ability of bitumen to contact and spread on the aggregate surface; that is to say, it can reflect the ability of asphalt-coated aggregates. Wettability can be calculated by measuring the contact angle of a liquid on a solid. Meanwhile, the quantitative index to evaluate the wettability is the spreading coefficient, which is defined as the amount of surface-free energy reduction in a solid when it loses its bare surface and forms new solid–liquid and liquid–air interfaces, expressed as follows:

(6) $$S_{a/s}=\gamma_{s}-\gamma_{as}-\gamma_{a}$$

where: $S_{a/s}$ is the spreading coefficient of asphalt (*a* denotes asphalt, *s* denotes aggregate), $\gamma_{s}$ is the surface-free energy of the aggregate, $\gamma_{a}$ is the surface-free energy of asphalt, and $\gamma_{as}$ is the interface of free energy between asphalt and aggregate.

 
  b. Adhesion

The work of adhesion is the free energy required to form a contact interface from the contact between a liquid and a solid. The work of adhesion between the asphalt and aggregate is expressed as follows:

(7) $$W_{as}=2\left(\sqrt{\gamma_{a}^{LW}\gamma_{s}^{LW}}+\sqrt{\gamma_{a}^{+}\gamma_{s}^{-}}+\sqrt{\gamma_{a}^{-}\gamma_{s}^{+}}\right)$$

where: $W_{as}$ is the adhesion work between the asphalt and aggregate, $\gamma_{a}^{LW}$, $\gamma_{a}^{+}$, $\gamma_{a}^{-}$ are the dispersion component, polar acid component, and polar alkali component of free energy on the asphalt surface, respectively, $\gamma_{s}^{LW}$, $\gamma_{s}^{+}$, $\gamma_{s}^{-}$ represent the dispersion component, polar acid component, and polar alkali component for the surface-free energy of aggregates, respectively.

 
  c. Flaking work

The flaking work can be expressed in detail as:

(8) $$W_{aws}=2\gamma_{w}+2\left(\begin{array}{c} \sqrt{\gamma_{a}^{LW}\gamma_{s}^{LW}}+\sqrt{\gamma_{a}^{+}\gamma_{s}^{-}}+\sqrt{\gamma_{a}^{-}\gamma_{s}^{+}}-\sqrt{\gamma_{a}^{LW}\gamma_{w}^{LW}}-\\ \sqrt{\gamma_{a}^{+}\gamma_{w}^{-}}-\sqrt{\gamma_{a}^{-}\gamma_{w}^{+}}-\sqrt{\gamma_{s}^{LW}\gamma_{w}^{LW}}-\sqrt{\gamma_{s}^{+}\gamma_{w}^{-}}-\sqrt{\gamma_{s}^{-}\gamma_{w}^{+}} \end{array}\right)$$

where: $\gamma_{w}$ is the surface-free energy of water, $\gamma_{w}^{LW}$, $\gamma_{w}^{+}$, $\gamma_{w}^{-}$ are the dispersion component, the polar acid component, and a polar base component of the surface-free energy of water.

 
  d. Energy ratio parameters

The adhesion between asphalt and aggregate increases with the increase in the spreading factor $S_{a/s}$ and work of adhesion $W_{as}$ and decreases with the increase in the work of spalling $W_{aws}$. As a result, researchers introduced the energy ratio parameter and the energy ratio parameter to synthesize the adhesion between the asphalt and aggregate [27]:

(9) $$E_{1}=\frac{W_{as}}{\left|W_{aws}\right|}$$

(10) $$E_{2}=\frac{S_{a/s}}{\left|W_{aws}\right|}$$

#### 2.3.3. AFM Test

AFM has been widely used to determine the nanomechanical properties of soft matter systems with success [28]. The continuously recorded force–distance curves reveal the adhesion properties between the tip and the most external sample surface and the energy dissipation [29,30]. In this paper, *Ra* was calculated using the Nanoscope analysis software 2.0 in the AFM technique as an indicator to assess asphalt surface roughness on a microscopic scale. The test was carried out using Dimension Icon AFM produced by Bruker, Germany, with the probe model RTESPA, the Quantitative Nanomechanical (QNM)-SADER method was used to calibrate the probe, and the elasticity constant of the probe was 5 N/m. The array of sampling points was set to 256 pixels × 256 pixels, the sampling frequency was set to 1 Hz, and the constant peak force was set to 1 nN, with a scanning range of 10 μm × 10 μm [31]. Three parallel tests under equal conditions were performed for each measurement. The AFM test temperature was about 20 °C at room temperature, and the asphalt specimens were prepared using the hot casting method.

## 3. Test Results and Discussion

### 3.1. Study on the Interfacial Spalling Performance of Recycled High-Viscosity Asphalt Aggregate under Dynamic Water Erosion Conditions

**Figure 1:**
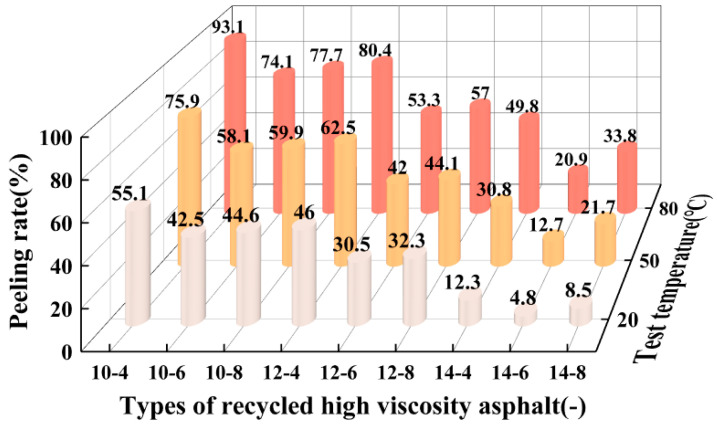
Peeling rate test results.

This paper uses the more common commercially available TPS high-viscosity modifiers that improve high and low temperatures as well as the fatigue resistance properties of asphalt and provide a sufficiently large bond between the aggregate and the aggregate. As the performance of high-viscosity asphalt decreased during the service period, it is important to use recycling agents to effectively restore its performance. In this paper, we used the Anshan Shuangcheng brand asphalt rejuvenator, which is a mixture of various resins, solvents, and multifunctional additives with low viscosity, stable properties, and rich in lightweight components. In addition, the asphalt viscosity is greatly affected by temperature; the asphalt viscosity at different test temperatures changes, which inevitably leads to changes in the adhesion of asphalt and the aggregate surface. Therefore, this paper simulated different weather temperature conditions on different recycled high-viscosity asphalt dynamic water flushing tests to spalling rates as an evaluation index to quantitatively analyze the effect of adhesion damage under different influencing factors, the test results of which are shown in Figure 1. This figure shows the recycled high-viscosity asphalt naming rules for the high-viscosity dosage-regeneration agent dosage.

As can be seen from Figure 1, there is a certain negative correlation between the flaking rate and high-viscosity agent dosage when the recycling agent dosage is certain; that is to say, the higher the high-viscosity agent dosage, the smaller the flaking rate of the reclaimed high-viscosity asphalt–aggregate, which indicates that the increase in the high-viscosity modifier could enhance asphalt adhesion. Taking the ambient temperature of 80 °C and the recycling agent dosage of 6% as an example, with the increase in a high-viscosity modifier dosage from 10% to 14%, the flaking rate of the recycled high-viscous asphalt–aggregate interface decreased from 74.1% to 20.9%, which was a decrease of up to 71.8%. For the reclaimed drainage pavement, whether the performance of aged asphalt could be effectively restored or not, the dosage of its reclaimer in aged asphalt had a significant impact. From the above figure, it can be seen that at a 20 °C, 50 °C, and 80 °C test temperature, when the amount of high-viscosity agent was constant, the influence of the amount of regenerant on the peeling rate showed a V-shaped distribution, indicating that the recycling agent dosage of 6% for recycling high-viscous asphalt–aggregate interface adhesion is the best. The reason for this may be that the insufficient dosage of the rejuvenating agent affects the recovery effect of high-viscosity asphalt performance, while the excessive dosage of a rejuvenating agent leads to an increase in the lightweight component of reclaimed asphalt, which has an unfavorable effect on the adhesion of asphalt. With the increase in the test temperature, the flaking rate of the recycled high-viscosity asphalt–aggregate interface gradually increased, indicating that the adhesion between asphalt–aggregate decreases with the increase in temperature. This is mainly due to the fact that temperature affects the viscosity of asphalt. The rheology of recycled high-viscosity asphalt gradually increases with increasing temperature, and the viscosity of asphalt becomes smaller, resulting in smaller adhesion between asphalt and asphalt and between the asphalt and aggregate, and in the process of dynamic water flushing, asphalt is more likely to be stripped from aggregate under the vibration of ultrasonic waves and cavitation, which ultimately leads to the destruction and spalling of the recycled high-viscosity asphalt–aggregate interface.

### 3.2. Research on the Adhesion Performance of Regenerated High Viscous Asphalt Based on Surface Energy Principle

#### 3.2.1. Surface Free Energy Measurement

Based on the surface-free energy introduction in Section 2.3.2 of this paper, the set of Equation (11) for calculating the surface-free energy of asphalt could be obtained by combining Equation (5) and the three known liquid surface energy parameters:

(11) $$\left\{\begin{array}{c} \gamma_{l1}\left(1+\cos\theta_{1}\right)=2\sqrt{\gamma_{l1}^{LW}\gamma_{a}^{LW}}+2\sqrt{\gamma_{l1}^{+}\gamma_{a}^{-}}+2\sqrt{\gamma_{a}^{+}\gamma_{l1}^{-}}\\ \gamma_{l2}\left(1+\cos\theta_{2}\right)=2\sqrt{\gamma_{l2}^{LW}\gamma_{a}^{LW}}+2\sqrt{\gamma_{l2}^{+}\gamma_{a}^{-}}+2\sqrt{\gamma_{a}^{+}\gamma_{l2}^{-}}\\ \gamma_{l3}\left(1+\cos\theta_{3}\right)=2\sqrt{\gamma_{l3}^{LW}\gamma_{a}^{LW}}+2\sqrt{\gamma_{l3}^{+}\gamma_{a}^{-}}+2\sqrt{\gamma_{a}^{+}\gamma_{l3}^{-}} \end{array}\right.$$

In the formula, $\sqrt{\gamma_{a}^{LW}}$, $\sqrt{\gamma_{a}^{-}}$, $\sqrt{\gamma_{a}^{+}}$ is the unknown quantity, substituting the surface-free energy of distilled water, formamide, and glycerol, and each component, solving the ternary system of equations, where the solution obtained is the surface-free energy component of asphalt, where the results of the calculations are shown in Table 3:

From Table 3, it can be seen that no matter which kind of asphalt is used, the value of the nonpolar component accounts for about 85% of the free energy of the surface of the asphalt, and the remaining polar component accounts for only 15%. Continuing to analyze the two parts of the polar component, it can be found that the alkaline component accounted for a very small percentage, only about 5%.

According to the same calculation method, the surface-free energy and components of basalt aggregate can be obtained, and the results are shown in Table 4.

From Table 4, it can be seen that the surface energy of basalt aggregate was high, as high as 233.01 mJ/m^2^, and when analyzing its surface energy component, it was found that the polar component accounted for the main part, close to 75.7%. In the polar component, it could be seen that the polar alkali component was as high as 491.7 mJ/m^2^, which is consistent with the nature of basalt as an alkaline aggregate.

#### 3.2.2. Evaluation of Interfacial Adhesion between Recycled High-Viscosity Asphalt and Aggregate

(1) Spreading factor

**Figure 2:**
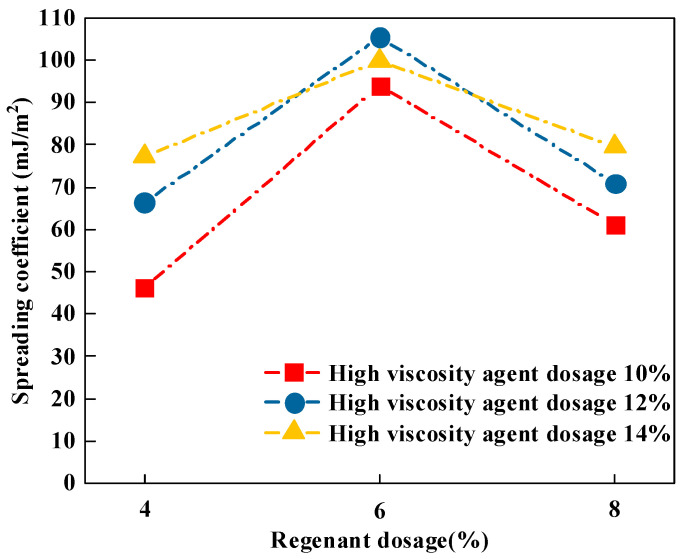
Test results of spreading coefficient calculation.

There is a significant correlation between the wettability of asphalt on the basalt aggregate and its adhesion effect. If the wettability of asphalt and aggregate is good and its spreading coefficient is high, asphalt can spread well on the aggregate surface, resulting in better adhesion performance between the two. On the contrary, if the wettability of asphalt and the aggregate is poor, and it is more difficult for asphalt to spread on the aggregate surface, then the spreading coefficient of the two is lower, and the adhesion performance of the two becomes worse. From an energetic point of view explanation, asphalt is in the process of wetting aggregate release energy where the size of the released energy is the spreading coefficient *S_a/s_*, the index of which can be quantitatively analyzed on the wettability of the abstract. In this study, the spreading coefficient of nine types of recycled high-viscosity asphalt on the surface of basalt aggregate is determined by Equation (6), and the calculation results are shown in Figure 2.

As can be seen from Figure 2, the case of the high-viscosity agent dosage was unchanged, with the increase in rejuvenating agent dosage, where the spreading coefficient between the recycled high-viscosity asphalt and basalt first increased and then decreased, all in the rejuvenating agent dosage of 6% to achieve the maximum value, which indicates that, at this time, the recycled high-viscosity asphalt and basalt aggregates had the best adhesion between the aggregates. The main reason for this is that, in a certain range, a greater dosage of the recycling agent increases the recycled high-viscosity asphalt of lightweight components in the content, improving the asphalt mobility and increasing the spreading coefficient. However, an excessive amount of the rejuvenating agent may form a gelatinous substance, resulting in a decrease in the spreading coefficient of recycled high-viscosity asphalt. In addition, with the increase in the high-viscosity agent, the spreading factor of reclaimed high-viscosity asphalt generally showed an increasing trend. However, the spreading coefficient of recycled high-viscosity asphalt with 12% high-viscosity agent at 6% recycling agent dosage is greater than that with 14% dosage, which might be due to the fact that the combination of the recycling agent and high-viscosity agent produces better wettability and fluidity at a specific dosage ratio.

(2) Adhesion function

**Figure 3:**
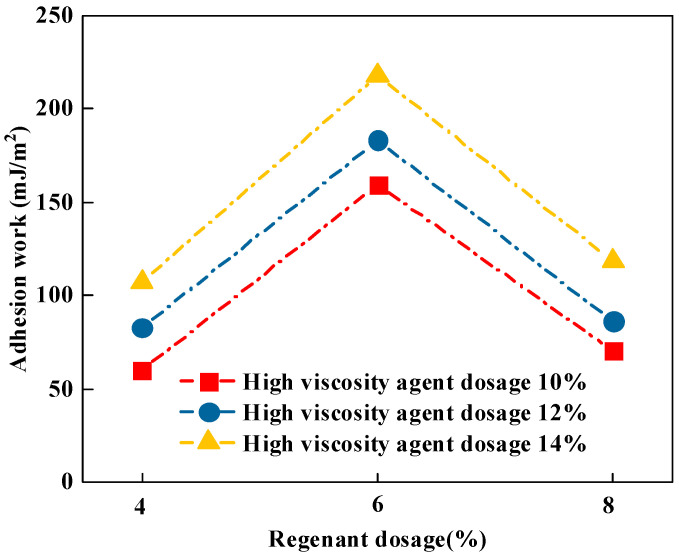
Adhesion work calculation results.

The energy required to strip asphalt from the asphalt–aggregate interface under dry and anhydrous conditions is the work of adhesion. The work of adhesion can be calculated from the parameters of surface energy and the fraction of bitumen as well as the aggregate. If the work of adhesion is higher, it represents the greater adhesion strength between the asphalt and aggregate. The work of adhesion between the asphalt and aggregate can be determined by Equation (7), which is calculated, as shown in Figure 3.

From Figure 3, it can be seen that similar to the rule of change in the spreading coefficient, when high viscous agent doping was certain, with the increase in rejuvenator doping, the adhesion function increased first and then decreased. The reason for this situation is that for the rejuvenating agent in a certain dosage range, the aging high-viscosity asphalt performance has a good recovery effect, the adhesion performance increases and the rejuvenating agent’s dosage further increases, leading to the rejuvenating agent in the asphalt diffusion not being uniform, resulting in the components of the miscibility appearing nonhomogeneous with damaged adhesion strength and the adhesion function becomes smaller [32]. In addition, under different conditions, the adhesion function of recycled high-viscosity asphalt becomes larger with the increase in a high-viscosity modifier dosage. The reason for this is that the increase in the dosage of a high-viscosity modifier can significantly increase the asphalt viscosity, thus improving asphalt’s adhesion.

(3) Flaking work

**Figure 4:**
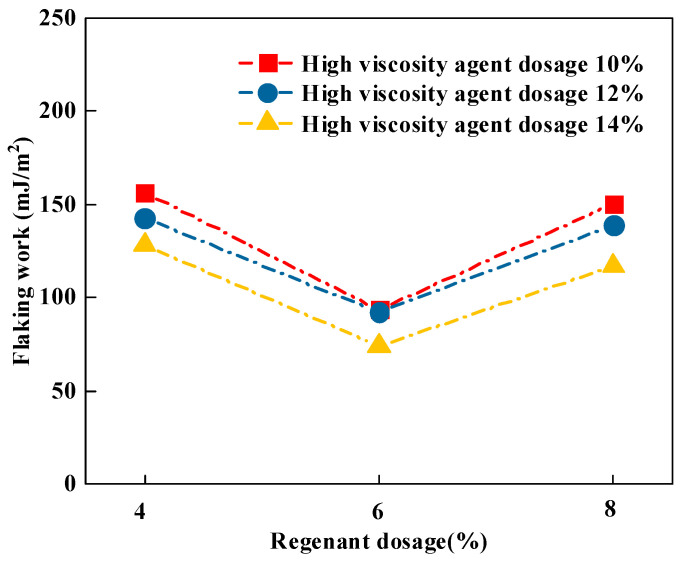
Calculation results of flaking work.

Water is one of the most important factors contributing to asphalt spalling, and the spalling function measures the likelihood of adhesion failure due to asphalt stripping from the aggregate in the presence of water. When water is present, there is a high likelihood that asphalt can be replaced by water at the asphalt–aggregate interface, resulting in the stripping of asphalt from the aggregate. From an initial asphalt–aggregate interface to a later water–aggregate interface, the overall surface energy of the system is reduced. The spalling work can be calculated by Equation (8), which is shown in Figure 4.

From Figure 4, it can be seen that the maximum value of the spalling work of the recycled high-viscosity asphalt and aggregate appeared in the case of 10% high-viscosity agent doping and 4% recycling agent doping for 156.01 mJ/m^2^ at the time that asphalt was most prone to spalling. The curve shows that there was an optimum value for the recycling agent dosage, and it was more difficult for the recycled high-viscous asphalt and aggregate at the inflection point to be spalled and damaged under the water environment. At a 6% recycling agent dosage, the spalling work of recycled high-viscosity asphalt and aggregate was the smallest, and the adhesion performance was the best. Further analysis can be found with an increase in the high-viscosity agent dosage where the absolute value of the spalling work between recycled high-viscosity asphalt and basalt aggregates gradually decreased, indicating better-recycled high-viscosity asphalt-–aggregate adhesion. This was due to the increase in the high-viscosity agent, which enhanced asphalt’s adhesion performance.

(4) Energy ratio parameters

**Figure 5:**
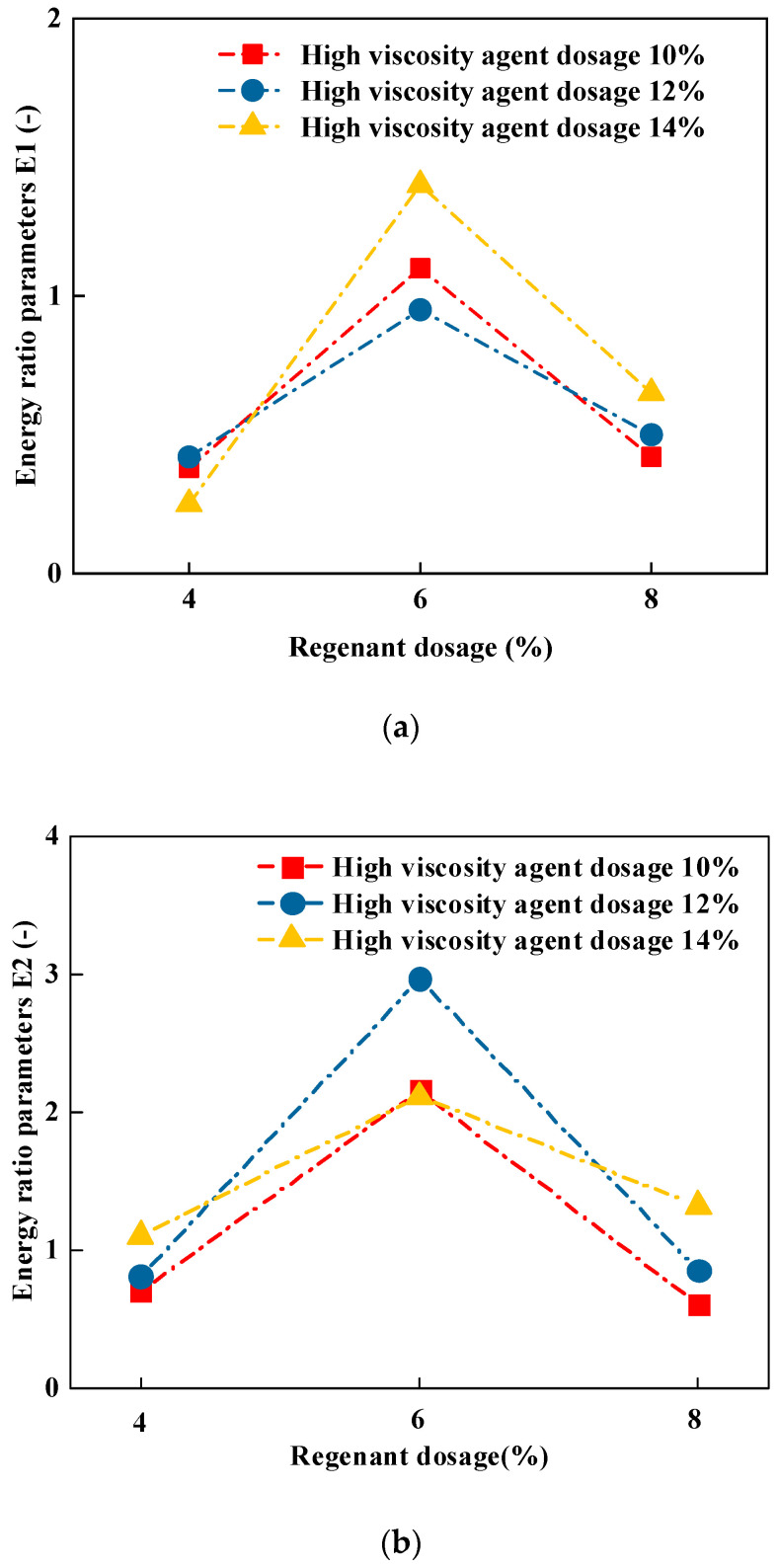
Calculation results of energy ratio parameters. (**a**) Calculation results of energy ratio parameter *E*_1_. (**b**) Calculation results of energy ratio parameter *E*_2_.

The spreading coefficient, the adhesion work, and the spalling work of different types of recycled high-viscosity asphalt on basalt are calculated above. The adhesion function is an indicator for evaluating the adhesion effect of asphalt–aggregate in the absence of water, while the spalling function is an indicator to evaluate the adhesion effect of asphalt–aggregate in the presence of water. However, in the actual use of the road, the two extreme states of extremely dry or sufficient water seldom occur but appear more in the exchange cycle of water and waterless conditions, so a single adhesion function or a single flaking function cannot reasonably characterize the actual adhesion effect of the recycled high-viscosity asphalt–aggregate interface. Therefore, in this paper, we need to comprehensively consider the adhesion performance indexes under aqueous and anhydrous conditions; therefore, two energy ratio parameters *E*_1_ and *E*_2_ are introduced for evaluation. The energy ratio parameter *E*_1_ is the ratio of the adhesion work to the flaking work, and the energy ratio parameter *E*_2_ is the ratio of the spreading coefficient to the flaking work, which are calculated according to Equations (9) and (10), and the results are shown in Figure 5.

From Figure 5a, it can be clearly seen that the energy ratio *E*_1_ of different recycled high-viscous asphalt was the smallest at 4% of the recycling agent dosage, which indicates that the adhesion between long-term aged asphalt and aggregate was extremely poor, and a sufficient recycling agent was needed to supplement the missing lightweight component of recycled high-viscous asphalt. In addition, under the condition of high viscous agent dosage determination, with the increase in the reclaiming agent, the energy ratio parameters, *E*_1_ and *E*_2,_ increased and then decreased, which indicates that the optimal reclaiming agent dosage selection should be paid attention to in the recycling of high-viscous aging asphalt. Through the energy ratio parameters of recycled high viscous asphalt, the high viscous agent dosage on the adhesion effect was not regular; the reason for this might be that the regeneration agent and high viscous agent on the recycled high viscous asphalt adhesion performance recovery effect had a synergistic effect.

### 3.3. Adhesion Characteristics of Recycled High-Viscosity Asphalt Aggregate Interface Based on AFM

#### 3.3.1. Microscopic Surface Feature Analysis

**Figure 6:**
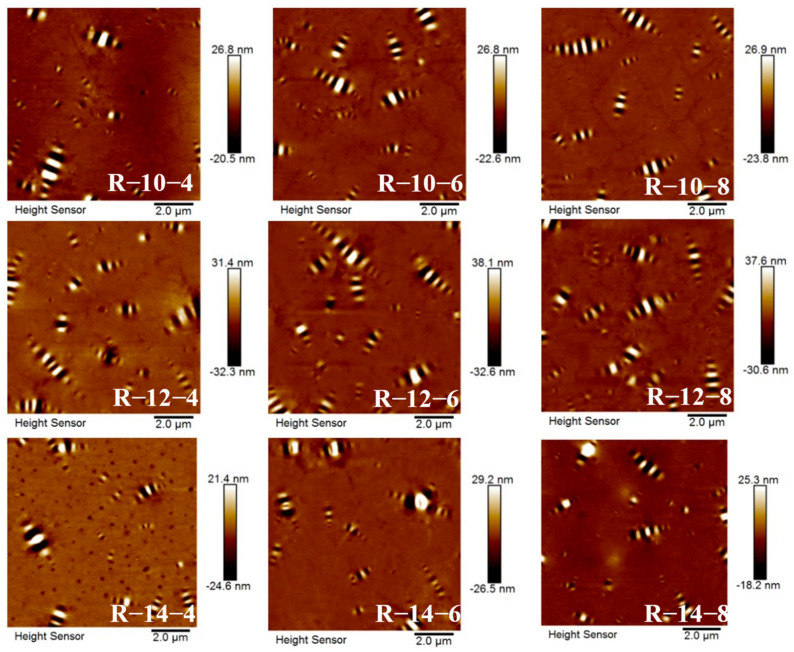
Surface micromorphology of different types of regenerated high viscous asphalt.

The two-dimensional surface morphology of the in-rejuvenated high-viscous asphalt with different high-viscosity agent and rejuvenator dosages is shown in Figure 6.

The 2D scanning image obtained by AFM clearly shows that the image consists of two main parts, namely the “bee-like structure” and the matrix phase. The area with alternating black and white bee-like shapes is called the “bee-like structure”, and the area around the “bee-like structure” is the matrix phase [33]. The number, maximum individual area, average area, total area, and area ratio of the bee-like structures in the 2D scans were counted using Image Pro-plus (IPP) software 6.0, and the results are shown in Table 5.

Analyzing the data in Table 5, it can be seen that with the increase in the high-viscosity agent, the number of honeycomb structures in the recycled high-viscosity asphalt roughly showed a decreasing trend, the total area of the honeycomb structure roughly showed an increasing trend, the maximum area of a single honeycomb structure showed a roughly decreasing trend, the average honeycomb structure area showed a gradually increasing trend, and the proportion of the total area also showed an increasing trend. This indicates that the adhesion capacity between the asphalt and aggregate was greatly enhanced with the increase in the high-viscosity agent dosage.

#### 3.3.2. Roughness

**Figure 7:**
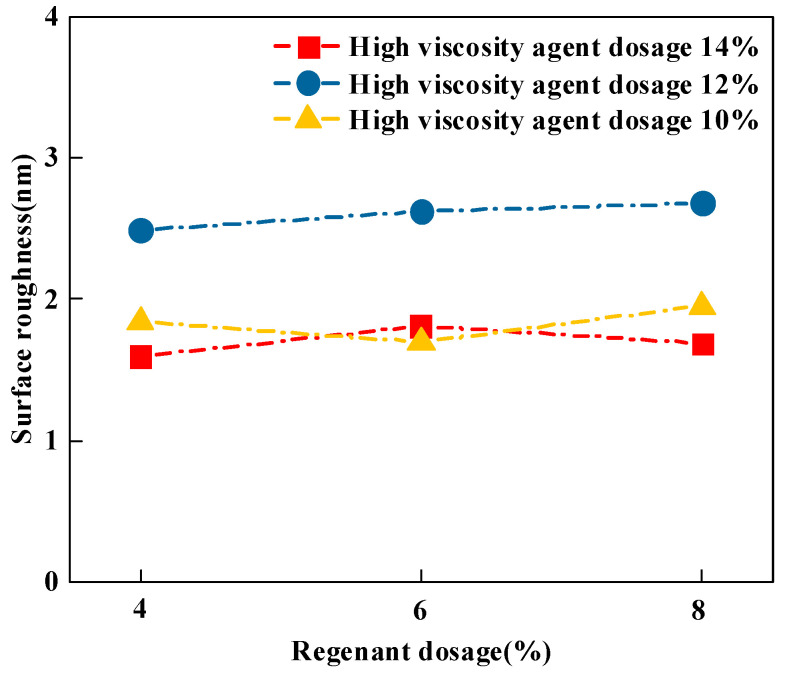
Surface roughness of different types of recycled high-viscosity asphalt.

Asphalt surface roughness is related to asphalt adhesion and its self-healing properties. The higher the surface roughness, the better the adhesion between the asphalt and aggregate [34]. The *Ra* results obtained from the AFM test are shown in Figure 7.

From Figure 7, it can be found that under the condition of 12% and 14% of high-viscosity agent doping, the surface roughness of the recycled high-viscosity asphalt increased from 5.7 nm and 3.91 nm to 6.24 nm and 4.35 nm, respectively, which increased by 0.54 nm and 0.44 nm, and the change fluctuation was very small. Under the condition of 10% high-viscosity agent dosing, the roughness decreased first and then increased with the increase in reclaimer dosing and finally changed by only 0.39 nm. Therefore, it was judged that the correlation between the reclaimer dosing and the roughness of the surface of reclaimed high-viscosity asphalt was not obvious. In addition, as a whole, the surface roughness was the largest at 12% high-viscosity agent dosing, and this roughness was similar at 10% and 14% dosing, which is obviously inconsistent with the actual situation. Therefore, this paper concludes that the use of roughness as an indicator for evaluating the adhesion performance of recycled high-adhesion asphalt needs to be further investigated.

#### 3.3.3. Adhesion

**Figure 8:**
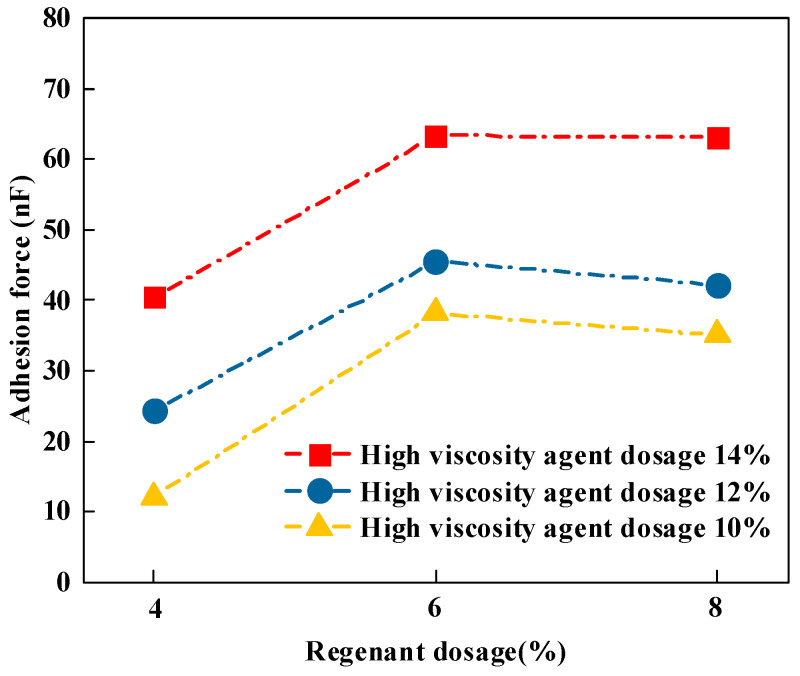
Adhesion of different types of recycled high-viscosity asphalt.

In order to further analyze the mechanical properties of recycled high-viscosity asphalt, the properties of its mechanical images were quantified using NanoScope Analysis software 2.0, and the statistical results of the adhesion of different recycled high-viscosity asphalt were obtained, as shown in Figure 8.

It can be seen from Figure 8, in the case of a certain amount of the high-viscosity agent, with the increase in recycling agent dosage, that the adhesion of recycled high-viscosity asphalt greatly increased and then slowly reduced. Taking the high-viscosity agent dosage R = 14% as an example with the recycling agent dosage from 4% to 6% in the process, the adhesion of the recycled high-viscosity asphalt increased by 56.1%; when the recycling agent increased dosage from 6% to 8% of the process, the adhesion of the recycled high-viscosity asphalt decreased by 0.81%. From this, the optimum rejuvenator dosage was judged to be 6% based on the adhesion. In the case of a certain amount of the recycling agent, the adhesion of recycled high-viscosity asphalt was positively correlated with the increase in high-viscosity agent dosage. The higher the viscous agent dosage, the denser the TPS high viscous agent in the uniform dispersion of matrix asphalt in the formation of a crosslinked network structure, showing a more stable state; at the same time, the network structure is also based on asphalt rheology’s ability to produce a damping effect, thereby improving asphalt adhesion, and further increasing the viscosity of asphalt.

### 3.4. Significance Analysis

The test results were analyzed using ANOVA using SPSS software 27 at a 95% confidence level to compare the significance of the effect of different factors on the adhesion performance parameters of the recycled high-viscosity asphalt, and the results are shown in Table 6. It was found that the modifier dosage and high-viscosity agent dosage had no significant effect on the energy ratio parameters *E*_1_ and *E*_2_ of the recycled high-viscosity asphalt, while almost all of the other adhesion performance parameters showed a significant effect. This suggests that the energy ratio parameter itself might not be applicable to the study of the adhesion characteristics of asphalt.

## 4. Conclusions

(1) In the ultrasonic equipment, simulated dynamic water scour test conditions, different high-viscous agent dosages of aging high-viscous asphalt with the addition of a rejuvenating agent, the rate of spalling on the aggregate surface with the increase in rejuvenating agent dosage decreased first and then increased. A 6% rejuvenating agent dosage of the aging high-viscous asphalt was used with the anti-stripping performance of the best restoration effect. In addition, simulating the adhesion performance test under different weather temperatures, it was found that the higher the temperature, the worse the anti-spalling performance of reclaimed high-viscous asphalt.
(2) Through the contact angle test, it was found that the spreading coefficient and adhesion work of recycled high-viscous asphalt on the surface of the basalt aggregate increased and then decreased with the dosage of the rejuvenating agent under anhydrous conditions, and the spalling work decreased and then increased under aqueous conditions, which is consistent with the conclusion of the kinetic water scour test. By calculating the adhesion performance indexes *E*_1_ and *E*_2_, it was found that the adhesion performance of recycled high-viscous asphalt increased and then decreased with the increase in the rejuvenating agent under the combined consideration of the state of water and no water, while the influence of high-viscous agent dosage on the adhesion effect was not regular, which could be due to the synergistic effect of the rejuvenating agent and the high-viscous agent in the recovery of adhesion performance of recycled high-viscous asphalt.
(3) The AFM test on different kinds of recycled high-viscosity asphalt “bee-like structure” morphology had some differences with the increase in high-viscosity agent mixing; its “bee-like structure” showed a number of reductions, the area of the trend increased, and there was no obvious change pattern as the amount of regenerant increased. There was no obvious connection between the roughness of recycled high-viscosity asphalt and its adhesion performance, and the pattern of change in adhesion was consistent with the results of the macro-adhesion performance test.

## Figures and Tables

**Table 1:** Technical indexes of base asphalt.

Evaluation Indicators	Test Results	Technical Requirement
25 °C pin man degree (mm)	69.0	60~80
Softening point (°C)	47.0	≥46
10 °C elongation (cm)	28.0	≥20
Solubility (%)	99.9	≥99.5
Density at 15 °C (g·cm^−3^)	1.02	-
Flash Point (°C)	272	≥260
135 °C rotational viscosity (Pa·s)	1.30	≤3
Film oven aging quality loss (%)	0.05	≤0.8
Film oven aging residual needle penetration ratio (%)	74.1	≥61
Film oven aging 10 °C residual elongation (cm)	10.0	≥6

**Table 2:** Basic performance parameter values for high-viscosity asphalt at different doses.

Type of Asphalt	Needle Penetration (0.1 mm)	Softening Point (°C)	10 °C Elongation (cm)
R-10-4	37.8	63.3	55.7
R-10-6	41.4	61.1	74.4
R-10-8	45.9	58.8	83.1
R-12-4	35.4	69.7	44.2
R-12-6	39.7	67.6	65.1
R-12-8	45.5	65.3	74.9
R-14-4	35.5	78.8	36.7
R-14-6	38.9	76.7	57.1
R-14-8	43.3	74.1	69.5

**Table 3:** Asphalt surface-free energy and its components (mJ/m^2^).

Recycled High-Viscosity Asphalt Types	Asphalt Surface Free Energy	Asphalt Dispersion Fraction	Asphalt Polarity Component	Asphalt Polar Acid Fraction	Asphalt Polar Base Fraction
R-10-4	23.95	21.96	1.99	0.23	4.32
R-10-6	28.85	25.84	3.01	0.49	4.65
R-10-8	25.84	25.14	0.70	0.05	2.52
R-12-4	25.29	21.51	3.77	0.50	7.14
R-12-6	32.67	28.02	4.65	1.02	5.30
R-12-8	38.19	33.89	4.30	1.47	3.13
R-14-4	47.02	39.26	7.76	3.54	4.25
R-14-6	51.01	44.53	6.47	3.44	3.05
R-14-8	57.60	49.66	7.93	5.21	3.02

**Table 4:** Basalt aggregate surface-free energy and fraction (mJ/m^2^).

Aggregates	Aggregates Surface Free Energy	Aggregates Dispersion Fraction	Aggregates Polarity Component	Aggregates Polar Acid Fraction	Aggregates Polar Base Fraction
Basalt	233.01	56.61	176.40	15.82	491.73

**Table 5:** Statistical results of “bee-like structure” data of 2D images.

Asphalt Type	Total Number	Total Area (μm^2^)	Individual Maximum Area (μm^2^)	Average Area (μm^2^)	Area Share (%)
R-10-4	58	5.381	1.228	0.118	5.4
R-10-6	44	4.886	1.062	0.132	4.8
R-10-8	51	5.022	1.218	0.158	5.1
R-12-4	50	6.878	0.568	0.118	6.8
R-12-6	54	6.848	0.608	0.156	6.8
R-12-8	48	6.751	0.512	0.132	6.8
R-14-4	46	7.522	0.880	0.150	7.6
R-14-6	37	9.384	0.734	0.172	9.4
R-14-8	32	8.411	0.800	0.174	8.8

**Table 6:** Results of variance analysis.

Factor	Sum of Squared Deviations	Degrees of Freedom	Mean Square Error	F-Value	*p*-Value
(1) Spreading coefficient					
Dosage of regeneration agent	2252	2	1126	25.33	0.005
Dosage of high-viscosity agent	553.8	2	276.9	6.229	0.059
(2) Adhesion work					
Dosage of regeneration agent	1.979 × 10^4^	2	9897	693.5	0.000
Dosage of high-viscosity agent	3988	2	1994	139.7	0.000
(3) Flaking work					
Dosage of regeneration agent	5548	2	2774	153.0	0.000
Dosage of high-viscosity agent	1142	2	571.0	31.51	0.003
(4) Energy ratio parameter *E*_1_					
Dosage of regeneration agent	1.560	2	0.780	4.274	0.174
Dosage of high-viscosity agent	3.552	2	1.776	4.402	0.021
(5) Energy ratio parameter *E*_2_					
Dosage of regeneration agent	4.568	2	2.284	17.20	0.010
Dosage of high-viscosity agent	0.280	2	0.140	1.054	0.428
(6) Surface roughness					
Dosage of regeneration agent	0.026	2	0.013	1.072	0.423
Dosage of high-viscosity agent	1.415	2	0.707	57.98	0.001
(7) Adhesion force					
Dosage of regeneration agent	1001	2	500.7	170.7	0.000
Dosage of high-viscosity agent	1153	2	576.6	196.6	0.000

## Data Availability

All the original data in this article can be obtained by contacting the corresponding author.
